# Can SARS-CoV-2 Infection Exacerbate Alzheimer’s Disease? An Overview of Shared Risk Factors and Pathogenetic Mechanisms

**DOI:** 10.3390/jpm12010029

**Published:** 2022-01-02

**Authors:** Chiara Villa, Eleonora Rivellini, Marialuisa Lavitrano, Romina Combi

**Affiliations:** School of Medicine and Surgery, University of Milano-Bicocca, 20900 Monza, Italy; e.rivellini@campus.unimib.it (E.R.); marialuisa.lavitrano@unimib.it (M.L.); romina.combi@unimib.it (R.C.)

**Keywords:** COVID-19, SARS-CoV-2, Alzheimer’s disease

## Abstract

The current coronavirus disease 2019 (COVID-19) pandemic, caused by severe acute respiratory syndrome coronavirus (SARS-CoV)-2, is affecting every aspect of global society, including public healthcare systems, medical care access, and the economy. Although the respiratory tract is primarily affected by SARS-CoV-2, emerging evidence suggests that the virus may also reach the central nervous system (CNS), leading to several neurological issues. In particular, people with a diagnosis of Alzheimer’s disease (AD) are a vulnerable group at high risk of contracting COVID-19, and develop more severe forms and worse outcomes, including death. Therefore, understanding shared links between COVID-19 and AD could aid the development of therapeutic strategies against both. Herein, we reviewed common risk factors and potential pathogenetic mechanisms that might contribute to the acceleration of neurodegenerative processes in AD patients infected by SARS-CoV-2.

## 1. Introduction

Coronavirus disease 2019 (COVID-19), caused by severe acute respiratory syndrome coronavirus-2 (SARS-CoV-2), continues to spread rapidly across the globe, becoming a devastating pandemic infection with growing mortality rates [1]. Although SARS-CoV-2 predominantly affects the respiratory system, increasing evidence reports a close relationship between COVID-19 and central nervous system (CNS) disorders, with more than 30% of hospitalized COVID-19 patients exhibiting neurological manifestations [2]. In line with this observation, magnetic resonance imaging (MRI) showed brain structural changes associated with COVID-19 in both surviving patients and non-survivors [3,4], confirming SARS-CoV-2’s involvement within the CNS [5]. However, whether the neurological symptoms represent a direct consequence of SARS-CoV-2 infection of brain cells or a result of systemic illness remains to be clarified [6]. A viral infection of human neurons has been suggested by the presence of viral RNA and/or protein in the brains of COVID-19 patients with neurological manifestations [7,8,9]. A neurochemical study reported that patients with severe SARS-CoV-2 infections exhibit high plasma levels of neurofilament light chain protein (NfL) and glial fibrillary acidic protein (GFAP), known as biochemical indicators of neuronal injury and glial activation [10], further supporting a direct link between SARS-CoV-2 brain infection and neurological disturbances. Additionally, both human and animal models demonstrated that the virus can directly invade the olfactory bulb [11] without a primary lung involvement, by the interaction between the virus S1 spike protein and angiotensin-converting enzyme 2 (ACE2), which is widely expressed in the glial cells and neurons [12]. However, the distribution of ACE2 in the human brain regions and cell types is quite heterogeneous. It is highly expressed in the choroid plexus of the lateral ventricle and central glial substance, while low expression was detected in the hippocampus. Regarding the cell-type distribution, ACE2 was found both in excitatory and inhibitory neurons, as well as in non-neuronal cells such as the oligodendrocytes, astrocytes, and endothelial cells. This evidence supports the hypothesis that brain infection by SARS-CoV-2 may promote CNS symptoms in patients with COVID-19, and suggests new potential routes for viral entry and propagation into the cerebral tissue [13]. SARS-CoV-2 may also infect the brain through a disrupted blood-brain barrier (BBB) that is often compromised in the aging brain, and neurodegenerative disorders, mainly in Alzheimer’s disease (AD) [14].

AD represents the most common form of dementia in the elderly population world-wide, and it is clinically characterized by neuronal loss in the hippocampus and cortical areas, leading to memory deterioration, behavioral changes, and cognitive decline. Neuropathological hallmarks include the presence of intracellular neurofibrillary tangles (NFTs), as well as parenchymal and vascular amyloid β (Aβ) deposits [15]. In the progression of the neuropathological changes observed in AD pathogenesis, a central role is played by neuroinflammation, attributed to activated microglia cells and the release of several cytokines [16]. Intriguingly, severe outcomes after SARS-CoV-2 infection in elderly individuals are often associated with a cytokine storm producing an excessive inflammatory and immune response, which may in turn accelerate brain inflammatory neurodegeneration [17]. Moreover, some COVID-19 patients could develop cognitive deficits after the primary infection [18], which can be partially explained by the virus-related exacerbation of the underlying brain pathology in elderly people [14]. One important issue is whether or not COVID-19 actually infects neurons, enters into neurons, or replicates within them, leading to a lytic cycle. Some data reviewed in [19] point to neuronal infection. Moreover, a recent study reported that SARS-CoV-2 spike S1 protein could facilitate the spreading of aggregated tau via the secretion of extracellular vesicles (EV) or direct cell-to-cell contact [20]. As SARS-CoV-2 is able to infect human neurons and use the neuronal machinery to replicate [21], the virus could lend its glycoproteins to neurons and EV, thus perpetuating the pathology. In addition to neuronal cells, astrocytes can also be infected by SARS-CoV-2, causing metabolic alterations that impair neuronal viability, contributing to neurodegeneration [22].

**Figure 1:**
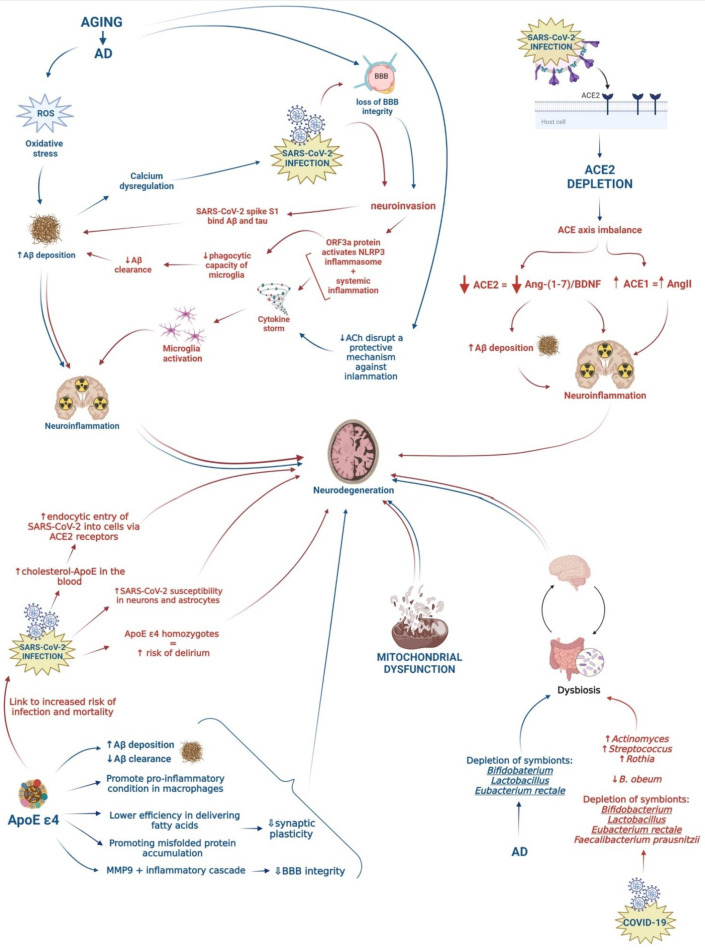
Schematic representation of possible pathogenetic mechanisms leading to neurodegeneration. Pathways activated by COVID-19 and AD are represented by red and blue arrows, respectively.

Given the high prevalence of AD individuals affected by COVID-19, this review aimed to elucidate common underlying etiological and risk factors that may contribute to the exacerbation of the neurodegenerative processes in AD patients infected by SARS-CoV-2. Understanding the relationship between COVID-19 and AD could aid in the detection of potential biomarkers for the early identification of COVID-19 in patients with a high risk of developing AD, as well as the management and development of novel therapeutic approaches for both diseases. Figure 1 shows the possible association between AD and SARS-CoV-2 infection by summarizing shared risk factors and potential underlying mechanisms, which are described in the following paragraphs (Figure 1).

## 2. Risk Factors and Potential Pathogenetic Mechanisms Shared by AD and COVID-19

### 2.1. Aging

Aging represents the primary risk factor for AD, with prevalence of the disease doubling every five years after age 65 [23]. Aging itself is also a well-known risk factor for severe disease and death in individuals affected by COVID-19 [24,25]. Epidemiological data from China showed that the case fatality ratio (CFR) of COVID-19 increases with age [24]; patients aged 59 years or older were at least 5 times more prone to die after the development of symptoms than younger ones [25]. Similarly, the CFR of COVID-19 in patients over 80 years was about 2-fold higher than the overall in Italy, the first country affected by the pandemic after China [26]. It has been postulated that aging may induce the generation of reactive oxygen species (ROS), exacerbate Aβ production, and increase neuroinflammation that contributes to the pathogenesis of both COVID-19 and AD [27,28]. Moreover, as aging is characterized by a gradual loss of the BBB integrity, elderly people could be more vulnerable to neuroinvasion during infection by SARS-CoV-2 [29].

It is well known that macrophages are affected by senescence, and thus older people respond weaker to SARS-CoV-2 infection due to the observation that macrophages have protective effects on the lungs during viral infection [30]. SARS-CoV-2 enters macrophages through the virus spike protein and induces them to produce high levels of senescence-associated secreted phenotype (SASP) factors, which contribute to Aβ accumulation, tau hyperphosphorylation, and the deposition of NFTs [31]. Therefore, aged macrophages and microglia may cause a different efficiency in response to pathology and infections.

### 2.2. Aβ Cerebral Deposition

Aβ is believed to be the key mediator of AD pathology and is one of the earliest brain AD-related molecular changes, starting several years before the onset of clinical symptoms [32]. Some evidence suggests that the potential increase of developing AD in COVID-19 patients may be related to Aβ. Intriguingly, a recent in vivo study provided data supporting the action for Aβ as an antimicrobial peptide [33]. The authors demonstrated that Aβ exerts a higher antimicrobial activity against the most common and clinically relevant microorganisms [34]. Therefore, it can be speculated that SARS-CoV-2 infection may stimulate or accelerate the Aβ accumulation in the brain, as part of an innate immune response, leading to AD. Another study demonstrated that the receptor-binding domain (RBD) of SARS-CoV-2 spike S1 protein can bind to several proteins, such as Aβ and tau, promoting their aggregation and further perpetuating the neurodegeneration process [34]. 

On the other hand, Aβ itself can facilitate the spread of SARS-CoV-2 infection through calcium (Ca^2+^) dysregulation. Integrating into the plasma membrane, the Aβ oligomers form pores, increasing the intracellular Ca^2+^ concentration [35] and thus contributing to neurodegeneration [36]. Interestingly, SARS-CoV-2 can disrupt Ca^2+^ pumps and channels, benefiting from an altered Ca^2+^ homeostasis in the AD brain for viral infection and its life cycle [37].

### 2.3. Angiotensin-Converting Enzymes

Angiotensin-converting enzymes (ACE), comprising ACE1 and ACE2, are key components of renin-angiotensin system (RAS), which antagonistically act by regulating the levels of angiotensin II (Ang II) and Ang-(1–7). In particular, ACE1 converts Ang I to Ang II and inactivates the vasodilator peptide bradykinin, whereas ACE2 is responsible for the cleavage of Ang II into smaller proteins such as Ang-(1–7), playing a role in vasodilation and antiproliferative effects [38]. Intriguingly, some inhibitors of ACE, by reducing Ang II levels, have been well documented to halt neurodegenerative disorders, including AD, by anti-inflammatory and antioxidant effects [39,40]. Ang-(1–7) proteins bind to Mas receptor (MASR) forming ACE2/Ang-(1–7)Mas axis, which is known to have a protective role in neurodegeneration in contrast to ACE1 and Ang II [38]. Through the activation of MASR, Ang-(1–7) regulated the activation of the PI3K/Akt/CREB/BDNF/TrKB pathway, thus inhibiting the inflammatory and oxidative stress events [41]. It is well known that the brain-derived neurotrophic factor (BDNF) plays an important role not only in neurogenesis and neurodevelopment, but also in normal mood behavior. As BDNF is released by the ACE2 enzyme, these findings raise the hypothesis of its involvement in the regulation of mental and neurological outcomes occurring during SARS-CoV-2 infection [42]. In ACE2 knockout mice, the deficiency of this enzyme resulted in an impairment of cognitive functions, probably in part due to a decreased BNDF and enhanced oxidative stress [43]. Conversely, the enhancement or overexpression of ACE2 lowered Aβ-related hippocampal pathology, ameliorated cognitive performance in the Tg2576 mouse model of AD-like brain amyloidosis [44,45,46] and improved Aβ-induced inflammatory responses [47]. Moreover, significantly diminished ACE2 activity and Ang-(1–7) levels were found in post-mortem AD brains and inversely correlated with Aβ and hyperphosphorylated tau levels [48]. In accordance, the plasma levels of Ang-(1–7) in AD patients were significantly decreased compared to the age-matched controls, suggesting their role as potential peripheral biomarkers for the disease diagnosis [49]. Similar evidence has also been reported in the senescence-accelerated mouse prone 8 (SAMP8), a naturally derived animal model of sporadic AD [50]. 

As already mentioned, ACE2 also serves as a receptor for SARS-CoV-2, thus allowing its entry to cells [29]. Hence, the binding of SARS-CoV-2 to ACE2 can result in the depletion of this enzyme, shifting the equilibrium towards ACE1/AngII and causing a further injury due to the above-described protective role of ACE2/Ang-(1–7)Mas axis against neurodegeneration [39,51]. This evidence supported the hypothesis that SARS-CoV-2 may inhibit the expression or activity of ACE2, leading to an exacerbation of cognitive impairment in patients with AD, and an augmentation of neurodegenerative processes. Consistent with this consideration, it has been found that advanced age is also associated with reduced ACE2 expression [52].

### 2.4. ApoE ε4 Allele

Apolipoprotein E (ApoE) is the main carrier of cholesterol in the CNS, where it is primarily expressed in both astrocytes and neurons [53]. This protein exerts neuroprotective effects in several brain functions, including neuronal plasticity and neurite outgrowth [54], as well as the regulation of Aβ neurotoxicity and clearance [55]. Microglia can also synthesize and release ApoE, and its secretion is finely regulated by neurons, suggesting microglia–neuron crosstalk [56]. While ApoE expressed in neurons and astrocytes seems to regulate the production of tau, microglia-secreted ApoE is involved in the control of microglial activation, and its production is induced during AD pathogenesis [57,58]. Among its three alleles (ε2, ε3 and ε4), individuals carrying the *APOE* ε4 allele are associated with increased risk and accelerated onset of AD by enhancing Aβ deposition into plaques and reducing its clearance from the brain [59]. Moreover, this allele impairs homeostastic functions of astrocytes and microglia, intersecting with changes that occur during normal aging to ultimately cause neurodegeneration and cognitive dysfunction [58]. *APOE* ε4 is able to promote pro-inflammatory conditions in macrophages and has lower efficiency in delivering essential fatty acids for neuronal membrane maintenance, as well as promoting misfolded protein accumulations, disrupting synaptic plasticity, and dendritic spine formation [60]. The *APOE* ε4 allele has also been involved in decreasing BBB integrity by activating the matrix-metalloproteinase-9 (MMP9) and inflammatory cascade [61]. Independent from pre-existing dementia or other comorbidities, a recent UK study reported that the *APOE* ε4 allele is linked to increased risk of infection and mortality due to COVID-19, although the biological mechanisms involved in this association remain to be elucidated [62]. Mechanistically, high levels of blood cholesterol by binding to the ApoE receptor are shown to improve the endocytic entry of SARS-CoV-2 to cells via ACE-2 receptors [63]. In an attempt to pinpoint the causal relationship between ApoE ε4 and COVID-19 susceptibility or severity, a study performed using human-induced pluripotent stem cells (hiPSCs) provided evidence that ApoE ε4 could lead to increased SARS-CoV-2 susceptibility in both neurons and astrocytes [64]. Compared to ApoE ε3 astrocytes, those with ApoE ε4 also exhibited a more exacerbated cellular response, including reactive astrocytes, enlarged size, and increased fragmentation of the nucleus—an indication of cell death, which may facilitate the progression and severity of COVID-19 [64]. 

Mainly in severe forms of COVID-19, impaired consciousness and delirium represented common clinical findings [65], and pre-existing dementia was related to high risk of severe SARS-CoV-2 infection as well as increasing mortality [66]. Intriguingly, ApoE ε4 homozygotes have an increased risk of delirium, in addition to dementia [67]. 

All these considerations suggested that SARS-CoV-2 infection may represent an aggravating factor for neurodegeneration in individuals with susceptible genetic variants. If further confirmed, APOE genotyping might help guide healthcare management of patients with comorbidities. 

### 2.5. Neuroinflammation and Microglia Activation

Another key feature of AD pathogenesis is represented by neuroinflammation, encompassing a series of inflammatory events in the CNS under pathological conditions [68]. The chronic inflammatory microenvironment in AD brains is reflected by high levels of cytokines, including interleukin-1β (IL-1β), IL-6 and tumor necrosis factor α (TNF-α) secreted by the activated microglia, the brain’s major innate immune cells [69]. Similarly, increased levels of IL-6 and TNF-α have been detected in the serum of AD patients compared to healthy individuals [70]. Moreover, it is well documented that systemic inflammation can affect cognitive functions and promote the progression of neurodegenerative disorders [71]. In this regard, the cognitive performance in AD individuals was found to be negatively correlated with IL-6 levels in plasma, suggesting a compromised cellular immunity in these patients [72]. In rodents, elevated levels of IL-1β in the brain impaired memory consolidation processes and cognitive functions, along with increased Aβ and NFT production [73]. The blockade of endogenous IL-1 [74] or knocking-out of IL-6 [75] resulted in an improvement in spatial memory and cognitive performance, as demonstrated by memory and learning behavior tests in in vivo models. 

In severe cases of COVID-19, SARS-CoV-2 infection can trigger systemic inflammation and a cytokine storm, leading to a significant increase of pro-inflammatory cytokines [76]. Indeed, significantly elevated levels of IL-1, IL-6, IL-8, IL-10, IP-10, and TNF-α have been found in the cerebrospinal fluid (CSF) of patients affected by COVID-19 [77,78,79]. Among pro-inflammatory cytokines, IL-6 is one of the most related to COVID-19, and is associated with a high risk of developing more severe diseases or mortality [79,80]. Similarly to IL-6, high levels of IL-1 are also predictors of a worse prognosis of SARS-CoV-2 infection [81]. These increased levels of cytokines could be partly due the SARS-CoV-2 open reading frame 3a (ORF3a) protein, which activates the innate immune signaling NOD-like receptor protein 3 (NLRP3) inflammasome with the consequent release of IL-1β, IL-6, and TNF-α [82,83]. After being activated, the NLRP3 inflammasome impaired the normal phagocytic capacity of microglia, reducing Aβ42 clearance in the brain [84]. Additionally, an in vitro study demonstrated that SARS-CoV-2 induces endothelial dysfunction at the BBB, impairing its integrity and function, thus enabling neuroinvasion [85]. Intriguingly, an intense microglia activation has been observed in post-mortem brains of patients with severe fatal COVID-19 [86]. Therefore, all this evidence might suggest that the massive inflammatory mediators released by the virus-induced systemic inflammation can enter the CNS through the damaged BBB in AD patients, thus amplifying the existing neuroinflammation and accelerating the neurodegeneration process [87,88]. 

Several studies have reported that the synthesis and release of IL-1β, TNF-α, IL-6, and IL-10 are mediated by acetylcholine (ACh) in a dose-dependent manner [89]. Synthesized by the enzyme choline acetyltransferase (ChAT) from acetyl-CoA and choline, ACh is an excitatory neurotransmitter of the CNS that plays important roles in learning and memory functions. In AD patients, a reduction in ChAT activity in the cerebral cortex has been found to be related to disease severity [90]. This results in a decrease of choline uptake and ACh release, leading to a presynaptic cholinergic impairment and thus affecting major brain processes, such as learning, memory, waking, and sleep [91,92]. Interestingly, an in silico study revealed that ACh is also involved in COVID-19-driven inflammatory responses. The authors reported that the high levels of prenatal choline in the mother can preserve the fetal brain’s development from the side effects of infection by SARS-CoV-2 [93]. On the basis of this evidence, it can be assumed that the decreased synthesis of ACh in AD brains may disrupt an important protective mechanism against inflammation, thus promoting a severe cytokine storm in COVID-19-affected patients. 

### 2.6. Oxidative Stress

Closely associated with the neuroinflammation, another critical factor involved in the development and progression of AD is oxidative stress, resulting from an imbalance between the generation of reactive oxygen species (ROS) and antioxidant defense mechanisms, leading to cell injury and neuronal death [94]. The combined effects of neuroinflammation and excess ROS production promote the aberrant accumulation of Aβ, thus contributing to AD [95]. Oxidative stress has a prominent role in innate immunity activity, and recent data have also reported its involvement in the pathogenesis of COVID-19, by perpetuating the cytokine storm cycle and exacerbating cell hypoxia [96,97]. After SARS-CoV-2 infection, ROS are overproduced as initiators of the toxic innate immune response against viruses [88]. Moreover, oxidative stress seems to be an important factor in aggravating disease severity in some patients with COVID-19, mainly associated with cytokine storm, pulmonary dysfunction, and viral sepsis caused by SARS-CoV-2 infection [98,99]. At the same time, the majority of people affected by both AD and COVID-19 are elderly, so they are more vulnerable to oxidative stress as it increases throughout the aging process. Given all these considerations, it can be postulated that SARS-CoV-2 infection may induce ROS-mediated oxidative modifications, perpetuating and amplifying oxidative stress in AD brains [88].

### 2.7. Mitochondrial Dysfunction

As reported above, ACE-2 is a key element for SARS-CoV-2 entry into the cell, and it is known that this protein exerts influences on mitochondrial activity. In particular, decreased ATP production, together with an abnormal activation of NADPH oxidase 4 in the mitochondria, are observed at low levels of ACE-2 [100]. NADPH oxidase 4 is important for ROS production, hence infected cells could be more sensitive to pathogens and more prone to go into apoptosis. An abnormal activity of mitochondria after the SARS-CoV-2 infection was also reported to be linked with the transmembrane serine protease 2 (TMPRSS2) from the virus acting on the estrogen-related receptor alpha which is an important mediator of mitochondrial biogenesis and function [100,101,102]. It was speculated that TMPRSS2 could also be involved in the gender differences of COVID-19 severity due to the fact that this protein can be induced by androgen but not estrogen, and resides on the mitochondria.

During the immune response, ATP production is reduced in favor of ROS production that, in turn, can damage the mitochondria in overwhelming amounts [103]. Once damaged, mitochondria could even release their contents into the cytosol, stimulating the release of IL-1β and IL-6 [103]. Increased levels of mitochondrial DNA (mtDNA) were normally observed in the cytoplasm of elderly people [104], and due to its role in promoting innate immunity and inflammation, this could contribute to the lethal outcomes seen in older COVID-19 patients [100].

People with a severe outcome of COVID-19 show high levels of ferritin [105]. Ferritin is produced as a storage molecule of iron when its intracellular concentration increases, and this iron/ferritin overload could either reduce oxygen consumption by mitochondria or disrupt glucose tolerance [106]. Interestingly, a cortical iron elevation is also observed as a feature of AD, and may contribute to the oxidative damage observed in AD brains [107].

The observation that 5′ and 3′ untranslated regions on SARS-CoV-2 contain mitochondrial localization signals [108] introduced the theory that the virus uses double-membrane vesicles budded by mitochondria to protect itself from ROS and host proteases [100]. 

Considering altogether, the detrimental effects of SARS-CoV-2 on mitochondrial activity, added to an already poorer baseline mitochondrial function in elderly individuals, could explain the worse outcome of the infection in this population. 

### 2.8. Gut Microbiota

A common factor observed in COVID-19 and neurodegeneration is an altered microbiota, known as dysbiosis. Several publications reported studies on both oral, fecal, and gut microbiome in COVID-19 patients. Zuo et al. observed the proliferation of opportunistic pathogens and a decrease of favorable commensal in the fecal microbiome of patients compared to healthy people [109]. More recently, Wu and colleagues found a decreased diversity in both the oral and gut microbiome of COVID-19 patients vs. healthy controls, and highlighted a strong association between the microbiome complexity and the disease severity [110]. In particular, they found elevated levels of *Streptococcus*, *Rothia*, and *Actinomyces* in COVID-19 feces and a depletion of *B. obeum*, confirming data observed by previous studies [109,111]. Moreover, they observed an enrichment in patients’ feces of *Granulicatella* and *R. mucilaginosa*, which are normally detectable in the respiratory tract flora.

An association between gut microbiota composition, levels of inflammatory markers, and cytokines was also confirmed recently by Yeoh at al. [112], who found an underrepresentation of gut commensals with known immunomodulatory potential (e.g., *Bifidobacterium*, *Eubacterium rectale* and *Faecalibacterium prausnitzii*) in COVID-19 patients. The authors commenting on their results underlined that it is still unknown whether this altered composition, which appears to be associated with greater severity of the disease, is directly involved in the disease or is related to clinical management of the patient (e.g., use of antibiotics) or to the individual immune status. A gut barrier dysfunction in COVID-19 patients was also reported by studying plasma [113]. The authors observed higher levels of gut permeability markers and the presence of microbes in the plasma of patients compared to controls. It is well known that the translocation of fecal microbiota into systemic circulation could be a key driver of immune response and inflammation [114,115,116,117], and thus may contribute to worsening COVID-19 outcomes [118].

A similar association between microbiome alteration and different levels of inflammatory proteins was previously reported for other diseases. For example, high relative levels of *E. rectale* in the gut was reported to be linked to reduced inflammation in AD [119]. Moreover, microbes involved in gut inflammation, oxidative damage and mucin-degradation are elevated in AD patients [120,121], and some bacterial species are able to directly participant in amyloidosis [122]. Studies using the 5XFAD mouse model of Aβ deposition expressing 5 familial AD mutations (FAD) suggested that depletion of gut bacteria results in an increased microglial uptake of Aβ [123]. In a small-scale clinical trial, the use of a mixture composed by *Bifidobacterium*, *Lactobacillus*, and *Lactococcus lactis* alleviated AD symptoms through modifications of the tryptophane metabolic pathway [124]. However, early probiotic treatments for AD led to contradictory results.

It was suggested that the diversity of gut microbiota could act as a common risk factor for neurodegeneration and COVID-19 infection, and that patients healed from COVID-19 would have higher odds to develop neurodegeneration [125]. 

## 3. Conclusions and Future Directions

The ongoing COVID-19 outbreak in late 2019 has caused a global pandemic with serious public health concerns. Apart from the well-known consequences to the respiratory system, increasing evidence reports that SARS-CoV-2 can invade the CNS, leading to severe neurological sequelae [126,127]. Once penetrated into the brain, the virus can cause neurodegeneration, demyelination, and cellular senescence, thus accelerating brain aging and potentially exacerbating the underlying neurodegenerative pathology [14]. 

Regarding AD, several mechanisms have been suggested to explain SARS-CoV-2-mediated neurological damage (Table 1), though its effects at the molecular and mechanistic levels remain only hypothetical or speculative, due to the absence of reliable post-mortem data on Aβ and NFTs in SARS-CoV-2-infected patients. AD and COVID-19 share many risk factors and pathogenetic mechanisms that may also partially explain the high incidence and mortality rate in people with AD. On the other hand, patients affected by AD could be more susceptible to contracting COVID-19. Preventive strategies to contain the SARS-CoV-2 spread, such as isolation or quarantine, negatively affect AD patients, increasing the risk of cognitive impairment due to a lack of social interaction [128]. Moreover, people living with dementia may be not able to follow recommendations from government authorities, such as sanitizing hands, covering the mouth and nose when coughing, and maintaining social distancing, partially due to their general cognitive impairment and short-term memory loss [129]. These patients are also more susceptible to circulating SARS-CoV-2, as they are frequently exposed to the virus during hospital care or required institutionalization and often suffer from pre-existing comorbidities, such as hypertension, diabetes mellitus and cerebrovascular diseases [130]. In this regard, the majority of patients with COVID-19 display severe coagulopathies, such as thrombotic microangiopathy and disseminated intravascular coagulation (DIC), probably due to hypoxic conditions or proinflammatory cytokines produced by infected cells [131]. Therefore, it is possible that microembolic events may contribute to cerebrovascular disease, which in turn can contribute to worsen AD pathology. However, it remains to be determined whether SARS-CoV-2 infection exacerbates cognitive decline in AD patients or triggers dementia in infected people, although many elements described here support this hypothesis. Additionally, it should be noted that some of pathogenetic mechanisms reported in this review are common to other neurodegenerative disorders, such as Parkinson’s disease and amyotrophic lateral sclerosis. In fact, novel research articles are continuing to report interesting results on other aspects. Among them, an overlap has been found between genetic risk factors for AD and severe COVID-19, such as single-nucleotide polymorphisms (SNPs) in oligoadenylate synthetase 1 (*OAS1*) [132] and bridging integrator 1 (*BIN1*) genes [133]. Single cell sequencing studies performed on brains of COVID-19 patients revealed that astrocyte and microglia cells show some pathological features shared with those observed in neurodegenerative disorders [134].

Although few studies addressing the relationship between COVID-19 and AD are currently available given the recent onset of the pandemic, their numerous shared links strengthen the necessity to assess neurological symptoms and implement preventive strategies to mitigate the risk of developing AD in SARS-CoV-2-infected people. Longitudinal follow-up studies of COVID-19 patients are needed to evaluate the long-term neurological effects of SARS-CoV-2 infection. Furthermore, large-scale retrospective analysis, in combination with preclinical studies, will be useful to fully understand the implications of SARS-CoV-2 infection for the development and progression of AD. Finally, these studies should be also implemented with cognitive impairment evaluation, blood and neuroimaging biomarkers evaluating inflammation, oxidative damage, or metabolic alterations, in order to assess the pathogenetic pathways shared by AD and COVID-19 that we reviewed. 

## Figures and Tables

**Table 1:** Summary of shared biological links between AD and COVID-19.

Pathway	Evidences for Mechanisms in AD	Evidences for Mechanisms in COVID-19	References
Aging	- Primary risk factor for developing AD	- Loss of BBB integrity facilitating SARS-CoV-2 neuroinvasion	[23,24,25,29]
Aβ deposition	- Key mediator of AD pathology and one of the earliest brain AD-related molecular changes - Increased intracellular Ca^2+^ concentration through formation of pores in cell membrane mediated by the Aβ oligomers	- Accelerated Aβ accumulation in the brain, as an antimicrobial peptide activating the innate immune response - Promoting the aggregation of Aβ and tau by the binding to SARS-CoV-2 spike S1 protein - Disruption of Ca^2+^ pumps and channels by SARS-CoV-2 infection	[32,33,34,35,37]
ACE axis imbalance	- Reduced ACE2 activity and Ang-(1–7) levels in post-mortem AD brains inversely correlating with Aβ and hyperphosphorylated tau levels - Decreased plasma levels of Ang-(1–7) in AD patients - Ameliorated cognitive performance by enhancing/overexpressing ACE2 - Protective role of ACE2/Ang-(1–7)Mas axis against neurodegeneration	- Mediating SARS-CoV-2 entry to cells by ACE2 as receptor for spike protein, resulting in enzyme depletion and the consequent shift of the equilibrium towards ACE1/AngI	[29,39,44,45,46,48,49]
ApoE ε4	- Increased risk of developing AD by enhancing Aβ deposition, promoting neuroinflammation, as well as disrupting synaptic plasticity and dendritic spine formation - Decreased BBB integrity by activating MMP9 and inflammatory cascade	- Enhanced endocytic entry of SARS-CoV-2 to cells via ACE-2 receptors through blood cholesterol associated to ApoE receptor - Increased risk of severe SARS-CoV-2 infection and mortality	[60,61,63,66]
Neuroinflammation and microglia activation	- Increased levels of IL-1β, IL-6 and TNF-α in AD brains and blood - Improved spatial memory and cognitive performance by blocking endogenous IL-1 or knocking-out IL-6 - Aggravating neurodegeneration by microglial activation - Impaired normal phagocytic capacity of microglia by activated NLRP3 inflammasome, resulting in reduced Aβ42 clearance in the brain - Reduction ChAT activity in the cerebral cortex related with disease severity and resulting in presynaptic cholinergic impairment	- Increased levels of IL-1, IL-6, IL-8, IL-10, IP-10, and TNF-α in the CSF of COVID-19 patients - Increased levels of IL-1 and IL-6 correlating with worse prognosis - Microglia activation in post-mortem brains of severe COVID-19 cases - Increased release of IL-1β, IL-6 and TNF-α partly due to SARS-CoV-2 ORF3a protein-mediated activation of NLRP3 inflammasome - ACh involvement in COVID-19-driven inflammatory response	[69,70,74, 75,77,78,79,82,83,84,86,90,93]
Oxidative stress	- Promoting the aberrant accumulation of Aβ in response to excessive ROS production	- Overproduction of ROS as initiators of the toxic innate immune response against SARS-CoV-2 - Aggravating disease severity if associated with cytokine storm, pulmonary dysfunction, and viral sepsis caused by SARS-CoV-2 infection	[88,95,98,100]
Mitochondrial dysfunction	- Increased cytoplasmic mtDNA levels promoting innate immunity and inflammation - Cortical iron elevation contributing to the oxidative damage in AD brains	- Promoting abnormal mitochondrial activity in the host cells mediated by TMPRSS2 - SARS-CoV-2 protection against ROS and host proteases mediated by double-membrane budded by mitochondria	[99,100,101,104,107]
Gut microbiota	- Altered gut microbiota composition associated with inflammatory markers - Elevated levels of microbes involved in gut inflammation, oxidative damage, and mucin-degradation - Involvement of some bacterial species in amyloidosis	- Altered gut microbiota composition, sometimes associated with inflammatory markers - Dysfunction in gut barrier related with worsened outcomes	[109,110,111,112,113,119,120,121,122]

ACE, angiotensin-converting enzyme; AD, Alzheimer’s disease; ApoE, apolipoprotein E; BBB, blood brain barrier; ChAT, choline acetyltransferase; COVID-19, Coronavirus disease 2019; CNS, central nervous system; CSF, cerebrospinal fluid; MMP9, matrix-metalloproteinase-9; mtDNA, mitochondrial DNA; NLRP3, NOD-like receptor protein 3; ORF3a, open reading frame 3a; ROS, reactive oxygen species; SARS-CoV-2, severe acute respiratory syndrome coronavirus-2; TMPRSS2, transmembrane serine protease 2.

## Data Availability

Not applicable.
